# Production of Soluble Receptor Activator of Nuclear Factor Kappa-Β Ligand and Osteoprotegerin by Apical Periodontitis Cells in Culture and Their Modulation by Cytokines

**DOI:** 10.1155/2019/8325380

**Published:** 2019-03-18

**Authors:** Miloš Duka, Mile Eraković, Zana Dolićanin, Dara Stefanović, Miodrag Čolić

**Affiliations:** ^1^Clinic for Stomatology, Military Medical Academy, Belgrade, Serbia; ^2^State University Novi Pazar, Serbia; ^3^Institute for Radiology, Military Medical Academy, Belgrade, Serbia; ^4^Medical Faculty of the Military Medical Academy, Belgrade, Serbia; ^5^University of East Sarajevo, Medical Faculty Foča, R.Srpska, Bosnia and Herzegovina

## Abstract

RANKL, a bone-destructive cytokine, and OPG, its osteoprotective counterpart, are expressed in periapical lesions (PLs), which represent hystopatological manifestations of apical periodontitis. However, their regulation in PLs has not been elucidated yet. Therefore, our aim was to study the production of RANKL and OPG and their modulation by pro- and anti-inflammatory cytokines in PL cell cultures. Isolated PL cells were cultured alone or with addition of TNF-*α*, IFN-*ϒ*, IL-17, IL-4, IL-10, and IL- 33, respectively. The levels of RANKL and OPG in supernatants were measured by ELISA. The proportion of CD3^+^ (T cells) and CD19+/CD138+ (B cells/plasma cells) within isolated PLs was determined by immunocytochemistry. The levels of RANKL were higher in cultures of symptomatic PLs compared to asymptomatic PLs and PLs with the dominance of T cells (T-type lesions) over B cells/plasma cells (B-type lesions). A higher proportion of osteodestructive processes (RANKL/OPG ratio > 1.0) were detected in symptomatic PLs. The production of RANKL was upregulated by IFN-*ϒ* and IL-17 and higher concentrations of IL-33. IL-10 and lower concentrations of IL-33 augmented the production of OPG. The addition of either RANKL or anti-RANKL antibody to the cultures did not modify significantly the production of OPG. In conclusion, this original PL cell culture model suggests that increased bone destruction through upregulated production of RANKL could be associated with exacerbation of inflammation in PLs with the predominance of Th1 and Th17 responses and increased secretion of IL-33. In contrast, IL-10 and lower levels of IL-33, through upregulation of OPG, may suppress osteolytic processes.

## 1. Introduction

Apical periodontitis is an opportunistic infection around the apical region, which is a consequence of spreading bacteria from the necrotic pulp [1]. This is a common disease in adults, with roughly one in three individuals affected [2]. The histopathological base of the disease consists of granuloma and radicular cysts, usually named periapical lesions (PLs). They are chronic processes, due to the inability of host defense mechanisms to eradicate the infection [3]. The pathophysiology of PL involves a complex host immune/inflammatory response to the bacteria and their products. The same mechanisms may also cause the destruction of soft and hard tissues surrounding the root apex [4]. PLs are characterized by the infiltration of the periodontal tissue with different inflammatory cells such as neutrophil granulocytes, T and B cells, plasma cells, macrophages, dendritic cells, mast cells, and other cells of the innate immunity [5]. The composition of infiltrating cells and the functional and phenotypic properties of both infiltrating and stromal cells depend on the activation status of PLs which is under control of a series of cytokines [3]. The histopathologic endpoint of PL is bone loss, which may occur to increase vascularization at the apex, thus blocking the infection in the root canal [6, 7].

Bone loss is caused by osteolytic activity of osteoclasts in which the receptor activator of nuclear factor kappa-Β ligand (RANKL) plays a crucial role. RANKL was initially identified as a cell membrane-bound ligand responsible for stimulation of osteoclast differentiation and bone resorption [8, 9], by mediating the cell-to-cell interaction between osteoblasts and osteoclast precursors. RANKL is also produced as a secreted ligand by osteoblasts, fibroblasts, and activated T and B cells as well as by the cells of the monocyte-macrophage lineage [10]. The metalloprotease-disintegrin TNF-*α*-converting enzyme mediates its cleavage into a soluble form. By activating its cognate RANK receptor on the surface of monocytes and macrophages, RANKL triggers the fusion of macrophages and their differentiation into mature osteoclasts with the bone resorption activity [11].

The action of RANKL is antagonized by osteoprotegerin (OPG). OPG is a soluble decoy receptor which blocks RANKL and, by preventing its interaction with RANK, inhibits osteoclast activation and subsequent bone resorption [12]. OPG is largely expressed by some epithelial cells, vascular endothelial cells, and lymphoid cells [13, 14]. The overall efficiency of RANKL on osteoclast formation and bone resorption is tightly coupled to the activity of OPG, as its natural inhibitor. Therefore, it is very important to study concomitantly the expression of these two molecules in sites of hard-tissue resorption, preferably as their relative RANKL/OPG ratio. The RANKL/RANK/OPG system is involved in pathogenesis of osteodestructive processes, including periodontitis and dentoalveolar development. Several papers described the expression of RANKL and OPG in both human and experimental animal PLs, by using immunohistochemistry, mRNA, or biofluid analyses, and the results have been summarized in a recent comprehensive review [15]. Generally, the review provides the evidence that higher RANKL expression and higher RANKL/OPG ratio are associated with periapical bone loss, but it does not give any conclusive information about their role as predictive markers, or their clinical significance. However, recent data showed that RANKL may play an immunoregulatory role since RANKL inhibition resulted in an unremitting proinflammatory response in experimental PLs, persistent high proinflammatory and effector CD4 responses, decreased migration of T regulatory cells (Tregs), and lower levels of IL-10 and TGF *β* [16]. All these data related to PLs are in contrast to a recent systematic review on biomarkers of alveolar bone resorption in gingival crevicular fluid, which showed that RANKL could be a central biomarker indicating osteoclastic activity and a diagnostic indicator for chronic periodontitis [17].

The expression of RANKL and OPG is under control of numerous factors, including cytokines, which play a crucial role in the regulation of immune/inflammatory reactions within PLs and are critical determinants of lesion outcome [4, 18]. In this context, proinflammatory cytokines, such as interleukin-1 (IL-1), IL-6, and tumor necrosis factor-*α* (TNF-*α*) as well as some chemokines, such as IL-8, orchestrate the recruitment and activation of innate immune cells, presumably neutrophil granulocytes and monocytes in the early inflammatory phase and T and B cells in the later inflammatory phase, respectively. A large number of clinical and experimental results suggest that T-helper 1 (Th1) cells, by producing interferon-*γ* (IFN-*γ*), are involved in the progression of PLs and bone destruction, whereas T-helper 2 (Th2) cytokines, such as interleukin 4 (IL-4), IL-5, IL-10, and IL-33, are associated with the humoral immune response and attenuation of tissue damage [3, 4, 6]. It seems that Th9 and Th22 pathways may also contribute to human and experimental periapical lesion stability [19]. IL-17 may play a role in exacerbation of inflammation in PLs [20] and stimulation of osteolysis [21]. On the other hand, Foxp3+CD4+CD25+ Tregs downregulate immune response, inflammation, and osteolysis in PLs. Their effect is predominantly mediated by transforming growth factor-*β* (TGF-*β*) and IL-10 [22–24].

The development of PLs is a dynamic process in which osteolytic and osteoprotective mechanisms are tightly balanced. However, up to now no one study investigated how a particular cytokine modulates these processes in PLs by acting on RANKL and OPG production, and this was a primary interest in our study.

To address these questions, we used an original approach by studying the production of RANKL and OPG in PL cell cultures. We described this cell-culture model in our previous paper and found it as very suitable to study the pathophysiological mechanisms involved in the progression and restriction of PLs [25]. Therefore, the concrete aim of this work was (1) to examine the production of RANKL and OPG by PL cells in culture, to determine their ratio, and to show the relationship between these parameters and clinical presentation of PLs, their size, and their lymphocyte composition within isolated PL cells; (2) to study the modulatory effect of proinflammatory cytokines (TNF-*α*, IFN-*ϒ*, and IL-17), anti-inflammatory cytokines (IL-4 and IL-10), and IL-33, a cytokine showing anti- and proinflammatory activity [26] on RANKL and OPG production by PL cells; and (3) to check whether the production of OPG by PL cells is modulated by RANKL.

## 2. Materials and Methods

### 2.1. Periapical Lesion Samples

Human PLs (*n* = 43) were extracted at the Department for Oral Surgery, Clinic for Stomatology, Military Medical Academy (MMA), Belgrade, Serbia, at the time of teeth extraction or apicotomy. The study was approved by the Ethical Committee of MMA in compliance with the Helsinki Declaration, followed by an informed consent from patients. The average age of the patients was 35 years (range: 21–65 years). The patients with malignant and autoimmune diseases, as well as patients on the immunosuppressive/immunomodulatory therapy, or those on the therapy of systemic modifiers of bone metabolism, were excluded. All the patients included had not been treated with antibiotics for one month before PL excision. PLs were radiographically diagnosed using the standard equipment for intraoral radiography (Carestream CS 2200 Roentgen apparatus; Carestream Dental, Atlanta, GA, USA) and extraoral radiography of the maxillofacial region (orthopantomography and dental cone beam computed tomography (CBCT); LargeV Instrument Corp. Ltd, Beijing, China). The size of radiolucent PLs on radiographs and tomographs was analyzed by adequate softwares, and smallest and largest diameters were measured. Three patients had two lesions on two different teeth. According to the presence or absence of clinical symptoms, PLs were classified as symptomatic (*n* = 22) or asymptomatic (*n* = 21).

The lesions were divided according to their size into small and large PLs. Small lesions (*n* = 18) were PLs whose mean diameter was less than 4.0 mm. The lesions whose mean diameter was higher than 5.0 mm were classified as large lesions (*n* = 25). No further division between specimens, regarding sex, age, etiology, or tooth type, was done. After extraction, PLs were immediately placed in a medium consisting of RPMI-1640 (Sigma-Aldrich, Taufkirchen, Germany) and antibiotics/antimycotic solution (Sigma-Aldrich) containing penicillin (100 units/ml), streptomycin (0.1 mg/ml), and amphotericin B (0.25 *μ*g/ml) and then transported to the laboratory.

Some PL cell cultures (*n* = 12) were used to study the modulatory effect of pro- and anti-inflammatory cytokines on RANKL and OPG production.

### 2.2. Isolation of Cells from PLs

The cells from PLs were isolated by a procedure which has been previously introduced by our research group [5, 25] with some modifications. Briefly, periapical tissue was placed in a Petri dish containing 1 ml RPMI-1640 medium and cut into 2-3 mm diameter pieces using a scalpel. The tissue was then digested for 15 min with 0.05% collagenase type IV (Sigma-Aldrich) and 0.02% DNAse (Sigma-Aldrich) in 5 ml RPMI-1640 medium in a cell incubator at 37°C. After that, the tissue was pressed through a stainless-steel mesh using a syringe plunger, filtered, and resuspended in RPMI-1640 medium containing 1 mM EDTA (Sigma-Aldrich). The remaining of the tissue was subjected to another round of digestion by using 0.05% collagenase/0.02% DNAse and 0.1% trypsin (Sigma-Aldrich) for 20 min. The released cells were pooled, washed twice by centrifugation in the RPMI medium containing 0.5 mM EDTA at room temperature (400 g for 10 min), and counted. The viability of cells, determined by Trypan Blue dye, was higher than 90%. After that, cytospins were prepared from each sample of cells using a cytocentrifuge (Schandon 4, Thermo Fisher Scientific, Waltham, MS, USA) on poly-L-lysine- (Sigma-Aldrich) coated glass slides. The cytospins were stained with May–Grünwald–Giemsa (Sigma-Aldrich) or used for immunocytochemistry.

### 2.3. Immunocytochemistry

For immunostaining, anti-CD3 monoclonal antibody (mAb) (Abcam, Cambridge, UK), anti-CD19, and anti-CD138 (mAbs) (both from Serotec, Oxford, UK) were used. Rabbit anti-mouse peroxidase-conjugated Ig was purchased from DAKO (Glostrup, Denmark). Cytospins were fixed with 2% pararosaniline (Sigma-Aldrich) for 2 min at room temperature, washed with phosphate-buffered saline (PBS) for 10 min, blocked with rabbit serum for 20 min, and washed with PBS. After that, cytospins were incubated for 60 min at room temperature with either anti-CD3 mAb, as a pan T cell marker, or the combination of anti-CD19/anti-CD138 mAbs, as markers of B cells and plasma cells, respectively, followed by washing with PBS. After that, the slides were blocked with 0.3% H_2_O_2_ in PBS for 20 min and then incubated with 1 : 50 dilution of polyclonal rabbit anti-mouse peroxidase-conjugated antibody. The immunoperoxidase reaction was developed with diaminobenzidine (Sigma-Aldrich). Controls were samples incubated with an irrelevant mAb, mouse anti-rat CD4 (OX-38) (Serotec), nonreactive with human cells. Cytospins were analyzed by light microscopy (Olympus, Hamburg, Germany). On each cytospin, at least 500 cells were counted. The percentages of positive cells were determined on the basis of total counted cells. Based on the predominance of T cells or B cells/plasma cells, PLs were divided into T-type and B-type lesions, respectively [3].

### 2.4. Cell Cultures

The cells isolated from PLs were cultivated in 96 wells, with round-bottomed plates (ICN, Costa Mesa, CA) (1 × 10^5^ cells/well, 200 *μ*l) in the complete culture medium consisting of RPMI-1640 medium supplemented with 10% fetal calf serum (FCS) (Sigma-Aldrich) and standard culture solutions of antibiotics [25]. Phorbol myristate acetate (PMA) (20 ng/ml) (Sigma-Aldrich) and Ca^2+^ ionophore (A 23187, 1 M) (Sigma-Aldrich) were used for cell stimulation [27]. After 24 h, the cell supernatants were collected, centrifuged, and frozen at −70°C until the levels of cytokines were determined. Certain cultures were used to study the modulatory effects of cytokines on RANKL and OPG production. The following cytokines were used: IFN-*γ;* (20 ng/ml), TNF-*α* (10 ng/ml), IL-17 (25 ng/ml), IL-4 (20 ng/ml), IL-10 (10 ng/ml), RANKL (10 ng/ml and 30 ng/ml), and IL-33 (1 ng/ml and 30 ng/ml). The concentrations of these cytokines were optimized in our previous experiments on peripheral blood mononuclear cells (PBMNC) and several PL cell cultures. All these cytokines were purchased from R&D (Lorton, VA, USA), except IL-33, which was obtained from BioLegend (San Diego, CA, USA). A neutralizing antibody to RANKL was obtained from R&D and used at the concentration of 2 *μ*g/ml.

### 2.5. Cytokine Assay

The concentrations of RANKL and OPG in culture supernatants were detected by using specific ELISA kits (Abcam) following the instructions of the manufacturer. The levels of cytokines were determined on the basis of the standard curve, constructed by known concentrations of these cytokines. The cut of values for RANKL and OPG was 10 pg/ml and 1 pg/ml, respectively.

### 2.6. Statistical Analysis

To assess whether the differences between the groups/samples were significant, either Wilcoxon signed-rank tests or Friedman test with Dunn's multiple comparison posttest was used, since the data did not follow the Gaussian distribution according to the Kolmogorov-Smirnov normality test. To assess whether RANKL and OPG levels correlate significantly, the Spearman correlation test was used and the values of *p* < 0.05 were considered to be statistically significant. The statistical analysis was carried out using GraphPad Prism (GraphPad Software, CA, USA).

## 3. Results

### 3.1. Production of RANKL in PL Cell Cultures in Relation to Clinical Characteristics of the Lesions and T/B Cell Predominance

In the first part of this work, the levels of soluble RANKL and OPG were determined in 43 different PL cell cultures. The results varied between samples such that the mean level of RANKL was 142.7 ± 116.2, the mean level of OPG was 91.8 ± 58.2, and the mean RANKL/OPG ratio was 2.5 ± 2.2. No correlation between RANKL and OPG levels was found (*r* = 0.30; *p* = 0.08). The proportion of PLs (62.8%) with active bone resorption processes (RANKL/OPG ratio > 1.0) was higher than the proportion of PLs (37.2%) in which bone resorption processes were suppressed (RANKL/OPG ratio < 1.0).

**Figure 1:**
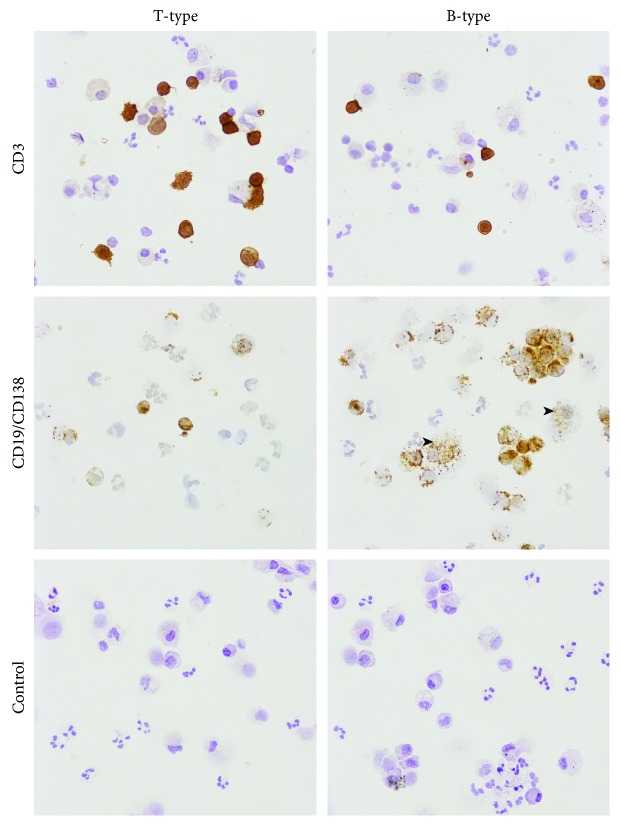
Immunocytochemical presentation of T-type and B-type periapical lesions. Cytospins of periapical lesion (PL) cells were stained with an anti-CD3 (marker of T cells) or a combination of anti-CD19 (B cell marker) and anti-CD138 (marker of plasma cells), as described in “Materials and Methods.” A representative sample of T-type PL (predominance of T cells over B cells/plasma cells) or a sample of B-type PL (predominance of B cells/plasma cells) over T cells is presented. Macrophages, which are positively stained with CD138 (intracytoplasmic granular staining), are excluded from the analysis, based on morphological criteria. Some of them are marked by arrows. Controls are stained by an irrelevant (anti-rat CD4) monoclonal antibody, nonreactive with human tissues. Magnifications: ×200.

**Figure 2:**
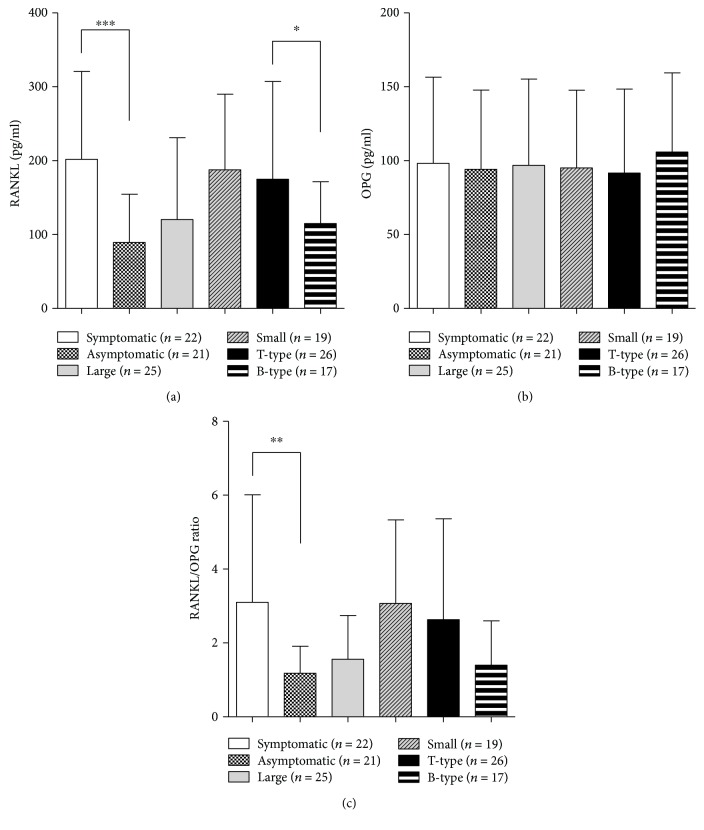
The levels of RANKL (a), OPG (b), and values of RANKL/OPG ratio (c) in PL cell cultures in relation to clinical characteristics of the lesions and T/B cell predominance. Periapical lesion (PL) cells were isolated from 43 PLs and cultivated for 24 hours as described in “Materials and Methods.” The levels of soluble RANKL and OPG in supernatants were measured by ELISA, and after that, the RANKL/OPG ratio was calculated. The differences between groups were tested by using the pair Student *t*-test. The statistically significant difference was marked on the graph. ^∗^*p* < 0.05, ^∗∗^*p* < 0.01, and ^∗∗∗^*p* < 0.001, as indicated.

The difference in the level of RANKL between symptomatic lesions (207.9 ± 119.8) and asymptomatic lesions (88.3 ± 67.7) was statistically significant (*p* < 0.001). No significant differences between large (122.4 ± 116.4) versus small (189.0 ± 112.3) lesions were found. The mean proportion of T cells was 18.6 ± 13.8, and the mean percentage of B cells/plasma cells was 16.3 ± 14.3. The number of PLs with the dominance of T cells over B cells/plasma cells (T-type lesions) was 27, whereas the number of PLs with the dominance of B cells/plasma cells over T cells (B-type lesions) was 16. An example of immunocytochemical images of the lesions is given in Figure 1. The difference in the mean levels of RANKL between T-type (189.6 ± 96.3) versus B-type lesions (116.2 ± 55.2) was statistically significant (*p* < 0.05) (Figure 2(a)). The differences in the levels of OPG between any of the examined groups were not statistically significant (Figure 2(b)). The difference in the RANKL/OPG ratio was significantly higher only between symptomatic (3.10 ± 2.91) compared to asymptomatic PLs (1.18 ± 0.73) (*p* < 0.05) (Figure 2(c)). The proportion of PLs with the RANKL/OPG ratio > 1.0 in symptomatic lesions (77.3%) was significantly higher (*χ*^2^ *F* = 4.044; *p* = 0.044) compared to the proportion of PLs with the same ratio in asymptomatic lesions (47.6%). No differences were found when the ratio was compared between the large versus small PLs, as well as T-type versus B-type PLs (Table 1).

### 3.2. Modulatory Effect of Pro- and Anti-Inflammatory Cytokines on RANKL and OPG Production in Culture of PL Cells

The second part of this work was related to the modulatory effect of pro- and anti-inflammatory cytokines on RANKL and OPG production, which was studied in 12 separate PL cell cultures. The proportion of PLs reflected generally their distribution in the whole group (the proportion of symptomatic and asymptomatic PLs was equal: *n* = 6; the proportion of small size PLs versus large PLs was 5/7; and the proportion of T-type versus B-type PLs was 7/5).

**Figure 3:**
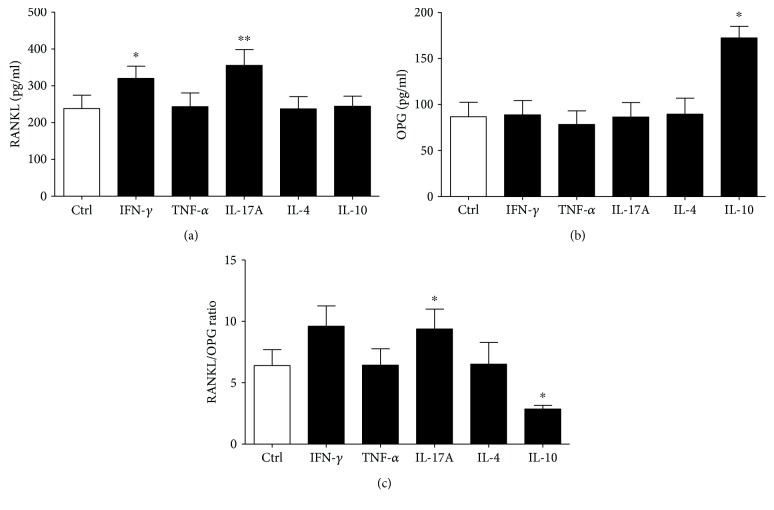
The levels of RANKL (a), OPG (b), and RANKL/OPG ratio (c) in PL cell cultures upon stimulation with pro- and anti-inflammatory cytokines. Periapical lesion (PL) cells were isolated from 12 PLs and cultivated for 24 hours with TNF-*α*, IFN-*ϒ*, IL- 17A, IL-4, and IL-10 as described in “Materials and Methods.” The levels of soluble RANKL and OPG in supernatants were measured by ELISA, and after that, the RANKL/OPG ratio was calculated. (a) Friedman test (*n* = 12, Friedman statistics 22.1, *p* = 0.0005) and Dunn's multiple comparison test, ^∗^*p* < 0.05 and ^∗∗^*p* < 0.01 vs Ctrl. (b) Friedman test (*n* = 12, Friedman statistics 15.94, *p* = 0.007) and Dunn's multiple comparison test, ^∗^*p* < 0.05 vs Ctrl. (c) Friedman test (*n* = 12, Friedman statistics 21.14, *p* = 0.0008) and Wilcoxon signed-rank test, ^∗^*p* < 0.05 compared to Ctrl.

IFN-*ϒ* and IL-17A augmented the production of soluble RANKL (*p* < 0.05 and *p* < 0.01, respectively) (Figure 3(a)). The level of OPG was only increased in the presence of IL-10 (*p* < 0.05) (Figure 3(b)), whereas IL-4 and TNF-*α* did not modulate the production of both RANKL and OPG (Figure 3(a) and Figure 3(b)). IL-17A increased the RANKL/OPG ratio, whereas IL-10 had the opposite effect (Figure 3(c)).

### 3.3. Modulatory Effect of IL-33 on RANKL and OPG Production in Culture of PL Cells

**Figure 4:**
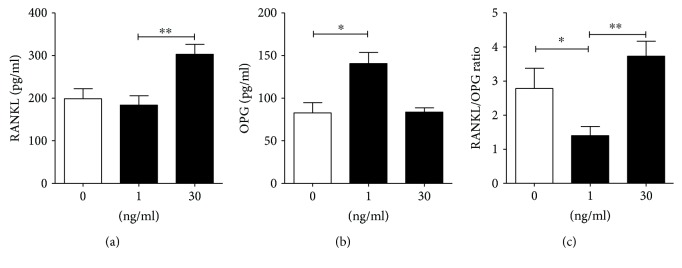
The levels of RANKL (a), OPG (b), and RANKL/OPG ratio (c) in PL cell cultures upon stimulation with IL-33. Periapical lesion (PL) cells were isolated from 12 PLs and cultivated for 24 hours with 1 ng/ml or 30 ng/ml of IL-33 as described in “Materials and Methods.” The levels of soluble RANKL and OPG in supernatants were measured by ELISA, and after that, the RANKL/OPG ratio was calculated. (a) Friedman test (*n* = 12, Friedman statistics 10.33, *p* = 0.0017) Dunn's multiple comparison test, ^∗∗^*p* < 0.01 as indicated. (b) Friedman test (*n* = 12, Friedman statistics 9.33, *p* = 0.0055) Dunn's multiple comparison test, ^∗^*p* < 0.05 as indicated. (c) Friedman test (*n* = 12, Friedman statistics 12.00, *p* = 0.0001) and Dunn's multiple comparison test, ^∗^*p* < 0.05 and ^∗∗^*p* < 0.01 as indicated.

The same 12 cultures were used to study the effect of IL-33 on RANKL and OPG production by PL cells. Based on preliminary results on PBMNC and PL cells, showing a dose-dependent effect of IL-33 on RANKL/OPG production and their ratio, we selected two doses (1 ng/ml and 30 ng/ml) of IL-33 for final experiments. As presented in Figure 4, lower concentrations of IL-33 upregulated OPG production and decreased the RANKL/OPG ratio (*p* < 0.05), whereas higher concentrations augmented RANKL production compared to the control (*p* < 0.01) and increased the RANKL/OPG ratio compared to the low dose of IL-33 (*p* < 0.01).

### 3.4. RANKL Does Not Modulate the Levels of OPG in Culture of PL Cells

**Figure 5:**
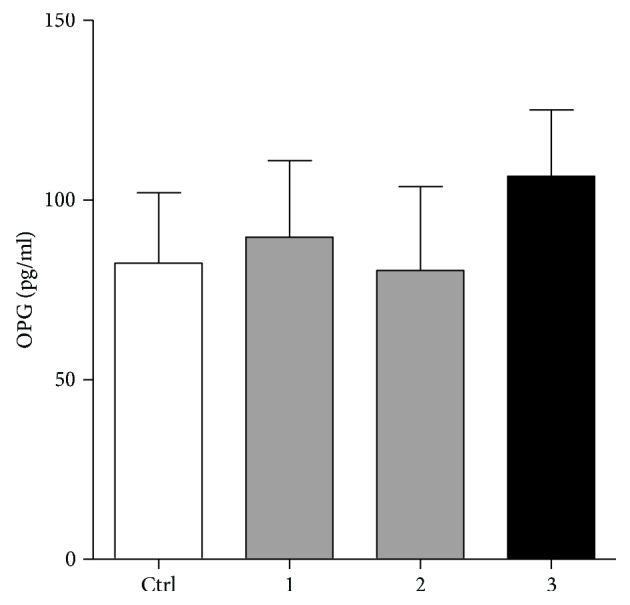
Modulation of OPG production in PL cell cultures by RANKL or anti-RANKL neutralizing antibody. Periapical lesion (PL) cells were isolated from 12 PLs and cultivated for 24 hours with 10 ng/ml (labeled as 1 on x axis), 30 ng/ml (labeled as 2) of RANKL, or anti-RANKL neutralizing antibody (2 *μ*g/ml) (labeled as 3) as described in “Materials and Methods.” The levels of OPG in supernatants were measured by ELISA, and after that, the RANKL/OPG ratio was calculated. All differences in relation to control (Ctrl) were not statistically significant (*p* > 0.05).

Finally, we tested whether the addition of exogenous RANKL modulates the production of OPG. According to mean levels of OPG (*n* = 12), it can be concluded that RANKL, at both of the two concentrations used (10 ng/ml and 30 ng/ml), did not modulate significantly the production OPG. A similar result was obtained by addition of a neutralizing anti-RANKL antibody (Figure 5).

## 4. Discussion

In this study, we showed that RANKL and OPG are produced in culture of cells extracted from the periapical tissue. This procedure has been introduced previously by our group with the aim to study the pathogenesis of human apical periodontitis [25]. Its advantage over other used approaches so far in humans, related to the expression of biomolecules associated with bone resorption/reparation processes, such as immunohistochemistry, mRNA expression, whole tissue homogenization, or biofluid collection, is the possibility to study the mechanisms involved, based on the manipulation with experimental conditions *in vitro*. In this context, the modulation of RANKL and OPG production by pro- and anti-inflammatory cytokines has been studied for the first time in this work.

The first *in vivo* demonstration of the involvement of RANKL and OPG in PLs came by immunohistochemical studies by using experimental pulpal exposure animal models. These and subsequent studies revealed a locally enhanced RANKL/OPG ratio in PLs and confirmed the implication of this molecular system in pathological periapical bone resorption, similarly as in other bone-destructive pathoses [15]. Several publications were related on RANKL and/or OPG in human PLs. Among them, Tay et al. showed the presence of RANKL in radicular cysts, by using an immunohistochemistry method [28]. Immunolocalization of RANKL was overlapped with staining for TRAP, a marker of osteoclasts. Sabeti et al. confirmed the presence of RANKL at the gene expression level in inflammatory PLs of undisclosed nature [29]. RANKL mRNA was also detected and semiquantified in periapical granulomas, whilst its expression was below detection limit in healthy periodontal ligament [30]. Expression of RANKL on inflammatory cells isolated from PLs was further investigated using flow cytometry. The results showed that monocytes (CD14+) and dendritic cells (CD83+) were the main producers of RANKL in granuloma [30]. By using an immunohistochemical study, Menezes and coworkers compared RANKL and OPG levels between apical granulomas and radicular cysts [31]. They found that the ratio of OPG^+^/total cells and RANKL^+^/total cells was higher in granulomas than in cysts, but the RANKL/OPG ratio did not differ between these two types of PLs. They also showed that various cell types expressed both RANKL and OPG, and the staining of macrophage-like cells (CD68^+^) was of the highest intensity. Another study demonstrated a significantly higher expression of RANKL mRNA levels in granulomas in comparison with cysts [32]. These results are in contrast to those published by Fan et al., who did not identify any difference between total RANKL or OPG protein levels or their ratio between granulomas and radicular cysts [33]. We also did not find the differences in these parameters between large- and small-size PLs. Although we did not classify our PL samples by histological criteria, radiological appearance was very suggestive that the majority of large size PLs resembled cysts, in contrast to small-size PLs. Considering RANKL and OPG expression and radiographic size of periapical granulomas (smaller or greater than 5 mm in diameter), Menezes et al. demonstrate a trend towards higher RANKL and lower OPG expression in smaller lesions, but similarly with our results, the differences were not statistically significant [34]. However, in their study the frequency of RANKL>OPG samples was higher compared to the group of larger lesions. No association of RANKL and OPG protein expression, detected by immunochemistry, with lesion size was also observed by Santos et al. [35]. Their study demonstrated the possible involvement of RANKL, TNF-*α*, IL-33, cathepsin K, and OPG in the development of radicular cysts and periapical granulomas, with emphasis on the highest immunoreactivity of cathepsin in cysts and TNF-*α* and OPG in granulomas. Based on these results, they supposed that OPG could determine the slower growth of granulomas compared to cysts due to its blocking activity against RANKL.

We showed that symptomatic lesions were characterized by higher production of RANKL and higher RANKL/OPG ratio, a phenomenon which has not been explored enough in previous studies. Our results are partly comparable with those published by Fan et al. who observed significantly more RANKL-positive cells in severely inflamed lesions compared to lightly inflamed counterparts. However, the RANKL/OPG ratio was statistically similar between inflammations graded as light, moderate, or intense [36]. The explanation why the production of RANKL is significantly higher in symptomatic PLs could be found by evaluating the association between high production of proinflammatory cytokines in symptomatic PLs [3] and RANKL expression. Among them, IL-1 and TNF-*α* have been shown to predominate both in the early phase of apical periodontitis and during the exacerbation, and both phases are characterized by the presence of clinical symptoms [3]. According to Kitaura et al., TNF-*α* acts directly to promote osteoclastogenesis, by increasing the expression of RANKL in macrophages and stromal cells [37]. Other studies suggest a direct effect of this cytokine on the bone resorption or through RANKL without modification of its expression. The resorption activity of osteoclasts generated by TNF-*α* in the absence of RANKL was critically dependent upon IL-1, which was expressed by the influence of TNF-*α* [38]. Zhang et al. found that TNF-*α* potently stimulated RANKL-induced osteoclastogenesis via coupling the RANK signaling pathway [39]. Further results indicated that IL-1 and LPS stimulate the production of osteoclasts through two parallel processes such as direct enhancement of RANKL and suppression of OPG expression, which is mediated by PGE2 production [40]. Kubota et al. demonstrated that TNF-*α* promotes the expression of OPG in synovial fibroblasts, predominantly through TNF-RI which may contribute to self-protection from the bone destruction [41]. We did not find any modulation of both RANKL and OPG production by TNF-*α*, suggesting that TNF-*α* has not a dominant effect on the expression of these bone-remodeling mediators in the mixture of the PL cell population, composed predominantly of inflammatory cells. It is interesting that we did not observe any significant modulation of OPG production by either addition of exogenous RANKL or its neutralization by anti-RANKL antibody, suggesting that, at least in this culture model, the expression of OPG is not under direct influence of RANKL. It is interesting that this phenomenon has not been examined in other cell systems. Another cytokine which is upregulated in symptomatic lesions is IL-17 [20]. IL-17^+^ T cells are important inducers of RANKL expression and can cause alterations in the RANKL/OPG balance [42]. In addition, RANKL is expressed by IL-17^+^ cells. However, a study showed that Th17 cells do not induce osteoclastogenesis in the absence of osteoblasts, which strongly suggests that RANKL expressed on Th17 cells alone is not sufficient to induce the differentiation of osteoclasts. This is partly dependent on a small amount of IFN-*γ* produced by Th17 cells, which could counterbalance the RANKL action [42]. It has been shown that IL-17 significantly enhances the expression of RANKL but also inhibits the expression of OPG in human periodontal ligament cells through mitogen-activated protein kinases and nuclear factor-*κ*B (NF-*κ*B) signals [43]. We showed that IL-17A augments RANKL, but not OPG secretion by PL cells, which is in accordance with the osteodestructive role of Th17^+^ cells. The increased number of activated T helper (Th) cells in PLs could be the reason why we detected a higher proportion of bone-destructive lesions (RANKL/OPG ratio > 1.0) in T-type lesions, compared with those with the predominance of B cells and plasma cells and in which humoral immune response prevails [3]. Among them are Th17^+^ cells. This is also in accordance with our findings that PL cells produce higher levels of RANKL in the presence of IL-17A.

Our previous results also showed that Th1 cells, which produce IFN-*ϒ*, are also numerous in PLs [25]. The role of IFN-*ϒ* in the pathogenesis of PLs and bone destruction is still controversial since IFN-*γ* can function as a pro- or antiresorptive cytokine [4, 32]. IFN-*γ* blunts osteoclast formation through direct targeting of osteoclast precursors but indirectly stimulates osteoclast formation and promotes bone resorption by stimulating antigen-dependent T cell secretion of RANKL and TNF-*α* [44]. The opposite results were published by Takayanagi et al. who showed that IFN-*ϒ* strongly suppresses osteoclastogenesis by interfering with the RANKL-RANK signaling pathway [45]. The analysis of the *in vivo* effects of IFN-*γ* in mouse models revealed that IFN-*γ* has both direct antiosteoclastogenic and indirect pro-osteoclastogenic properties *in vivo*. Our findings, which showed that IFN-*γ* stimulates the production of RANKL by PL cells in culture, are in accordance with those showing that Th1 cells, through induction of RANKL, play a direct role in bone resorption in Th1-dominant diseases [46].

However, our explanations about the dominance of osteodestructive processes in T-type lesions are simplified because other Th subsets (Th2, Th9, Th22, and Tregs) as well as different subpopulations of CD8^+^ T cells are also present in PLs. Th2 cells, by producing IL-4, IL-5, and IL-13, are believed to be associated with the humoral immune response in which anti-inflammatory processes dominate [3]. However, we found that only half of B-type lesions belong to the group of bone antiresorptive PLs (RANKL/OPG ratio < 1.0). It has been shown that IL-4 directly prevents osteoclast precursors from differentiating into osteoclasts in a signal transducer and activator of transcription (STAT) 6-dependent manner [47]. In addition, IL-4 suppresses RANK expression but enhances OPG expression in osteoblastic cells [47]. We did not show any modulatory effect of IL-4 on RANKL and OPG production in PL cell cultures, suggesting that most inflammatory cells from the PLs respond differently to IL-4 than osteoclasts do.

The only subpopulation of T cells which strongly inhibits inflammation and bone destruction is the Treg subset. Tregs produce IL-10 and TGF-*β*, which downregulate the production of RANKL and increase the production of OPG [48]. IL-10 directly inhibits osteoclast precursors by suppressing RANKL-induced NFATc1, c-Fos, and c-Jun expression [49]. Our results, showing an increased production of OPG and decreased RANKL/OPG ratio in PL cell cultures, support previous findings. However, we did not detect any significant change in the production of RANKL.

An interesting finding in our study was related to the dual effect of IL-33 on RANKL and OPG production. We showed for the first time that lower doses of this cytokine augment OPG production in PL cells, in contrast to higher doses which augment RANKL. It is known that IL-33 belongs to the IL-1 family and is closely related in structure to IL-18 and IL-1*β*. The cytokine is synthesized as a biologically inactive precursor and then cleaved by the enzyme caspase-1 to be secreted as active mature forms. IL-33 stimulates target cells by binding to its ST2 receptor which is followed by activation of NF-*κ*B and MAPK pathways via identical signaling events to those observed for IL-1*β*. IL-33 is also a nuclear factor abundantly expressed in high endothelial venules. The major targets of IL-33 *in vivo* are tissue-resident immune cells such as mast cells, group 2 innate lymphoid cells (ILC2s), and Tregs. However, it also acts on many other cells including Th1 cells and CD8 cells. Initially, IL-33 was proven as a potent stimulator of Th2 immune response (allergy diseases), but we know now that IL-33 is a crucial immune modulator with pleiotropic activities in type 2, type 1, and regulatory immune responses, playing important roles in allergic, fibrotic, infectious, and chronic inflammatory diseases [26]. The knowledge of the role of IL-33 in periodontal diseases is relatively scarce, and the results are contradictory. In this context, Malcolm et al. demonstrated that the expression of IL-33 and ST2 was elevated in gingival tissues from patients with chronic periodontitis as compared with healthy tissues. Similarly, IL-33 expression was observed to be higher in periodontal tissues of *Porphyromonas gingivalis*–infected mice. IL-33 upregulated the expression of RANKL in such mice. In contrast, administration of OPG, by targeting RANKL, abrogated periodontal destruction [50]. Therefore, IL-33 could act as a proinflammatory cytokine with osteodestructive consequences. Similarly, Velickovic et al. showed higher expression of IL-33 and ST2 in periapical granulomas and radicular cysts when compared to healthy periapical tissues, suggesting that IL-33/ST2 signaling may be involved in periapical inflammation and tissue fibrosis [51]. Araujo-Pires et al. did not find any significant difference in the expression of IL-33 between active (RANKL>OPG) and inactive (RANKL<OPG) human periapical granulomas [52]. However, the opposite results were found in ST2 knockout mice in which deletion of ST2 signaling augmented the RANKL/OPG ratio in PLs. The number of CD3^+^ RANKL+ cells along with TRAP+ osteoclasts was higher in PLs of ST2^–/–^ mice compared with wild-type (WT) mice, whereas the percentages of CD3^+^ OPG+ cells were higher in WT mice. In addition, ST2 deletion increases inflammatory bone loss, which was associated with enhanced Th1/Th17 cell-mediated periapical immune responses and increased osteoclastogenesis [53]. Based on all these results, our findings suggest that during exacerbation of inflammation in PLs, IL-33 could potentiate bone resorption through increased production of RANKL. However, during resolution of inflammation, low secretion of IL-33, through augmented production of OPG, could act as a bone-protective cytokine.

In conclusion, PL cells in culture produce a significant amount of both RANKL and OPG, so we propose this model as a useful alternative to study mechanisms involved in the pathogenesis of apical periodontitis. Some of them, which were examined in this work, suggest that both bone resorptive and bone-protective processes are present in all stages of PL development and are finely balanced by cytokines involved in PL pathogenesis.

## Figures and Tables

**Table 1:** RANKL/OPG ratio in PL cell cultures depending on the clinical presentation of PLs and T/B cell predominance.

RANKL/OPG ratio	Sy *n* (%)	As *n* (%)	∑ *n* (%)	*χ* ^2^ *F*	*p*
<1.00	5 (22.7)	11 (52.4)	16 (37.2)	4.044	*0.0443*
>1.00	17 (77.3)	10 (47.6)	27 (62.8)		

	Large *n* (%)	Small *n* (%)	∑ *n* (%)		
<1.00	10 (40.0)	6 (33.3)	16 (37.2)	0.1991	0.6555
>1.00	15 (60.0)	12 (66.7)	27 (62.8)		

	T-type *n* (%)	B-type *n* (%)	∑ *n* (%)		
<1.00	7 (26.9)	9 (52.9)	16 (37.2)	2.978	0.0844
>1.00	19 (73.1)	8 (47.1)	27 (62.8)		

Sy: symptomatic lesions; As: asymptomatic lesions.

## Data Availability

The data used to support the findings of this study are available from the corresponding author upon request.
